# A lipidomic and metabolomic signature of a very low-carbohydrate high-fat diet and high-intensity interval training: an additional analysis of a randomized controlled clinical trial

**DOI:** 10.1007/s11306-023-02071-1

**Published:** 2023-12-23

**Authors:** Lukas Cipryan, Vit Kosek, Carlos J. García, Tomas Dostal, Kamila Bechynska, Jana Hajslova, Peter Hofmann

**Affiliations:** 1 Department of Human Movement Studies and Human Motion Diagnostic Centre, The University of Ostrava, Ostrava, Czech Republic; 2 Department of Food Analysis and Nutrition, Faculty of Food and Biochemical Technology, University of Chemistry and Technology, 16628 Prague, Czech Republic; 3 Institute of Human Movement Science, Sport and Health, Exercise Physiology, Training and Training Therapy Research Group, University of Graz, Graz, Austria

**Keywords:** Carbohydrates, Lipids, Exercise, Diet, Lipidomics, Metabolomics

## Abstract

**Introduction:**

Regular physical activity and dietary variety are modifiable and influential factors of health outcomes. However, the cumulative effects of these behaviors are not well understood. Metabolomics may have a promising research potential to extend our knowledge and use it in the attempts to find a long-term and sustainable personalized approach in exercise and diet recommendations.

**Objective:**

The main aim was to investigate the effect of the 12 week very low carbohydrate high fat (VLCHF) diet and high-intensity interval training (HIIT) on lipidomic and metabolomic profiles in individuals with overweight and obesity.

**Methods:**

The participants (N = 91) were randomly allocated to HIIT (N = 22), VLCHF (N = 25), VLCHF + HIIT (N = 25) or control (N = 19) groups for 12 weeks. Fasting plasma samples were collected before the intervention and after 4, 8 and 12 weeks. The samples were then subjected to untargeted lipidomic and metabolomic analyses using reversed phase ultra-high-performance liquid chromatography coupled to high-resolution mass spectrometry.

**Results:**

The VLCHF diet affected plasma lipids considerably while the effect of HIIT was unremarkable. Already after 4 weeks of intervention substantial changes of plasma lipids were found in both VLCHF diet groups. The changes persisted throughout the entire 12 weeks of the VLCHF diet. Specifically, acyl carnitines, plasmalogens, fatty acyl esters of hydroxy fatty acid, sphingomyelin, ceramides, cholesterol esters, fatty acids and 4-hydroxybutyric were identified as lipid families that increased in the VLCHF diet groups whereas lipid families of triglycerides and glycerophospholipids decreased. Additionally, metabolomic analysis showed a decrease of theobromine.

**Conclusions:**

This study deciphers the specific responses to a VLCHF diet, HIIT and their combination by analysing untargeted lipidomic and metabolomic profile. VLCHF diet caused divergent changes of plasma lipids and other metabolites when compared to the exercise and control group which may contribute to a better understanding of metabolic changes and the appraisal of VLCHF diet benefits and harms.

**Clinical Trial Registry number:**

NCT03934476, registered 1st May 2019 https://clinicaltrials.gov/ct2/show/NCT03934476?term=NCT03934476&draw=2&rank=1.

**Supplementary Information:**

The online version contains supplementary material available at 10.1007/s11306-023-02071-1.

## Introduction

The traditional and usually applied “one size fits all” approach for exercise and diet interventions may not be suitable for a long-term and sustainable effective way in primary and secondary prevention of various chronic noncommunicable diseases such as obesity, diabetes, neurodegenerative diseases, cardiovascular diseases or cancer (Agostoni et al., 2021). Therefore, in the context of nutrition science, the research focus has turned to a personalized or precision nutrition (PN) approach. This is a complex effort to understand the health effects of the relationships among genetics, microbiome, antibiotic and probiotic use, metabolism, food environment and physical activity, as well as economic, social, and other behavioral characteristics (Rodgers & Collins, 2020). Since metabolism plays a central role in nutrition and the rapid development in molecular analysis, metabolomics have been established as an important analytical tool in human nutritional studies (Ulaszewska et al., 2019) and attempts for PN.

Metabolomics, along with genomics, transcriptomics, and proteomics, is a part of novel omics technologies that have shown a promising research potential (Sébédio, 2017). Metabolomics identifies and quantifies low molecular weight molecules in biofluids, tissues or cells (Müller & Bosy-Westphal, 2020). The metabolomics analysis results in lists of molecules (the metabolome) describing the metabolic phenotype of individuals (metabotype) (Sébédio, 2017). However, metabolic profiling approaches to identify markers of dietary exposure and the interpretation of metabolomic data are still in the early developmental phase (Müller & Bosy-Westphal, 2020; Ulaszewska et al., 2019). Also, metabolomic “fingerprinting” should not be confused with a mechanistic understanding (Müller & Bosy-Westphal, 2020).

Nutrition, physical activity and exercise play a crucial role in prevention and treatment of multiple chronic diseases (Pedersen & Saltin, 2015). Systemic and metabolic diseases such as type 2 diabetes, high blood pressure, obesity or metabolic syndrome, have risen globally to an epidemic burden, all of them with typical different pathophysiological patterns. These disorders increase the risk for many other chronic diseases, including cardiovascular diseases and cancer. A large body of evidence showed that avoiding unhealthy eating patterns, particularly high amount of processed or poor quality foods (Ludwig et al., 2023) and regular physical activity support people achieving and maintaining health, and reduce the risk of chronic diseases (Ehrman et al. 2019; Riebe, 2018). In order to treat such diseases, targeted individual exercise and diet programs need to be developed to overcome the actual “One-size fits all” strategy.

The optimal macronutrient proportion in humans’ nutrition has never been established in detail. Thus, it is still unclear whether a high or low carbohydrate intake is the best for human health in modern times. Contrary to usually “beliefs” of a dominant carbohydrate intake to be beneficial, a carbohydrate restricted diet was recently shown to represent an alternative promising dietary strategy to prevent various pathological conditions involving inflammatory and autoimmune pathways (Lennerz et al., 2021; Miller et al., 2020; Youm et al., 2015). A significant decrease of waist circumference was presented in individuals with overweight and obesity who participated in a combined exercise and low carbohydrate ketogenic diet intervention (Lee & Lee, 2021; Miller et al., 2020). This effect was observed also despite maintaining the participants daily intake of energy and levels of physical activity (Gram‐Kampmann et al., 2022). The possible explanation of a body mass reducing effect of such a carbohydrate restricted diet was suggested an increased total energy expenditure after approximately 2.5 week of lower-carbohydrate intake (Ludwig et al., 2020). However, a satiety effect of a carbohydrate restricted high fat diet, which may cause a total energy decrease, must be considered (Ludwig et al., 2023).

High-intensity interval training (HIIT) is a training alternative to the traditional moderate-intensity continuous endurance training. HIIT is defined as periods of intense activity separated by low-intensity breaks. HIIT can improve the fitness level to a greater extent than continuous exercise despite a reduced training volume (Milanović et al., 2015). Such a superior effect of HIIT has been shown also to improve metabolic health (Cassidy et al., 2017; Jelleyman et al., 2015), vascular function, cardiovascular risk factors (Grace et al., 2018; Sawyer et al., 2016), oxidative stress, inflammation, and insulin sensitivity (Ramos et al., 2015). However, similar responses to HIIT and moderate-intensity continuous exercise were presented for cardiometabolic adaptation (De Nardi et al., 2018; Gillen et al., 2016). When an acute effect of HIIT and moderate-intensity continuous exercise matched for strain were analyzed from the metabolomic perspective, a comparable metabolic and hormonal response was shown despite substantial differences in work-bout intensities (Moser et al., 2015; Zafeiridis et al., 2016).

Regular physical activity and dietary variety are modifiable and influential factors of health outcomes. However, the cumulative effects of these behaviors are not well understood. We aimed to investigate the effect of a 12 week very low carbohydrate high fat (VLCHF) diet and HIIT on lipidomic and metabolomic profiles in individuals with overweight and obesity. This work extends our earlier findings, which focused primarily on body composition, exercise performance and standard biochemical outcomes (Cipryan et al., 2021, 2022).

## Methods

### Parent study

It was a randomized, controlled, four-arm, parallel exercise and/or dietary intervention study (ClinicalTrials.gov: NCT03934476), with the primary aim of examining the VLCHF and HIIT effects on body composition and cardiorespiratory fitness (Cipryan et al., 2021). Data were collected in Ostrava, Czech Republic. There were 91 participants included into four study groups and they completed a 12 week experimental period. Participants were randomly allocated to one of the four study groups: (1) HIIT and habitual diet, (2) very low-carbohydrate, high-fat diet (VLCHF) and habitual physical activity (no regular exercise training), (3) VLCHF diet and HIIT and (4) control (habitual diet and physical activity, no regular exercise training). Dual-energy X-ray absorptiometry (DXA) and graded exercise tests to volitional exhaustion were used for body composition and cardiorespiratory fitness (CRF) assessments, respectively.

To obtain measures of no intervention, a control group was utilized. Participants in the control group were advised not to change their habitual diet and physical activity regime. Therefore, no diet advice was provided.

We utilized the infrastructure of this trial to conduct a pre-planned ancillary study focused on lipidomic and metabolomic analysis. We analysed blood samples following a 3 h fast before the experimental period (T_0_) and after 4, 8 and 12 weeks (T_1_, T_2_ and T_3_, respectively). The participant set used for the primary and secondary analyses was identical with the participant set presented in this metabolomics study (intention-to-treat analysis).

### Participants

Participants were randomly allocated to four study groups: (1) HIIT and habitual diet, (2) very low-carbohydrate, high-fat diet (VLCHF) and habitual physical activity (no regular exercise training), (3) VLCHF diet and HIIT, (4) control (habitual diet and physical activity, no regular exercise training) (Cipryan et al., 2021, 2022). Inclusion criteria were age 20–59 years, non-smokers, overweight/obesity (BMI 25.00–40.00 kg/m^2^), no specific sports training or regular exercise (low physical activity), no excessive alcohol intake, willingness to accept random assignment, no evidence of liver, renal, metabolic and cardiopulmonary disease and diseases contraindicating physical activity, no cancer, no psychiatric illness, no pregnancy or breast-feeding, not on any specific diet, PAR-Q pass, body weight stable for the last 2 months and not on a weight loss plan, no hypoglycaemic, lipid lowering, antihypertensive or psychiatric medications as well as medications known to affect body weight or energy expenditure. Participants had no previous experience with the VLCHF diet and HIIT. All study participants provided written informed consent. The study design was approved by the local University Ethics Committee.

### High-intensity interval training (HIIT)

Before the start of the intervention, the participants in both HIIT and VLCHF + HIIT groups received a detailed instruction on the HIIT program. Participants were told to complete 3 sessions per week. Two HIIT sessions were completed during weeks 4, 8 and 12 when the participants visited laboratory, one session was home based. Each HIIT session started and finished with 5 min of slow walking. HIIT was composed of series of 3 min of a high intensity walking (Borg´s scale RPE 18–19) followed by 3 min of low intensity walking (RPE 9–11). There were 4, 6 and 8 high intensity intervals set up for the first, second and third 4 week period, respectively. Therefore, the total session time increased from 31 to 43 min and finally 55 min during each 4 week period. Training intensity was monitored with a Polar M430 watch (Polar Electro, Oy, Finland). Training data were uploaded to Polar Flow and regularly analysed by an experienced researcher.

### Dietary intervention

Participants in the HIIT only and the control group were asked to maintain their habitual dietary intake without restriction. The VLCHF diet was defined as allowing no more than 50 g of CHO per day (Feinman et al., 2015). The diet included no specific caloric goal. However, the participants in the VLCHF groups were advised to compensate for the total energy decrease caused by CHO intake restriction by increasing their natural non-trans fat intake (e.g., cream, butter and olive and coconut oil). The target for protein intake was recommended 1.5 g/kg lean body mass and, unlike the strict CHO restriction, participants were asked just to get as close as possible. The use of all sweetened and grain-based products was strongly limited. The recommended food included whole food sources such as meat, vegetables, non-sweetened products, full-fat dairy items, nuts and seeds. Detailed dietary advice was provided by a dietitian before and during the study (on request or at least once a month). A handbook was given to participants containing food lists, guidelines for estimating macronutrient amounts, and sample recipes. All foods and quantities consumed were recorded daily in all study groups beginning from seven days before the intervention period (www.kaloricketabulky.cz). Alcoholic beverages were restricted during the intervention period and dietary supplements were not permitted 1 month prior to and during the intervention, while caffeinated beverages were restricted only before the laboratory sessions.

### Chemicals and materials

Methanol and 2-propanol were supplied by Merck (Darmstadt, Germany). Ammonium acetate, ammonium formate, formic acid and acetic acid were obtained from Sigma-Aldrich (Prague, Czech Republic). Click Fit 2 mL Eppendorf tubes were purchased from TreffLab (Degersheim, Germany); 2 mL cryovials and autosampler vials were purchased from Labicom (Olomouc, Czech Republic).

#### Sample preparation

The samples were prepared by mixing 50 μL of plasma with 150 μL MeOH. After vortexing, 500 μL methyl tert-butyl ether (MTBE) was added, and the mixture was shaken for 15 min. Subsequently, 150 μL of deionized water was added to form a two-phase system. After vortexing for 30 s, the mixture was deproteinized and centrifugated at 10,000 rpm (10,621×*g*) for 10 min at room temperature. The resultant supernatants were lyophilized and stored in an − 80 °C freezer if needed for later use. The freeze-dried lipid residues were resuspended in 2-propanol/methanol/deionized water (65:30:5, v/v/v) and used for subsequent analysis. For the metabolomic experiment 50 uL of plasma were mixed with 150 uL acetonitrile to precipitate proteins, the mixture was then centrifuged at 10,000 rpm and the supernatant collected in a new vial and evaporated to dryness on a SpeedVac (Labconco) instrument. Before analysis, the dried extract was resuspended in H_2_O:acetonitrile (1:1).

#### Instrumental conditions

Both lipidomic and metabolomic analysis used an Infinity 1290 (Agilent) UHPLC coupled to the 6560 Ion Mobility Q-TOF LC/MS (Agilent) with an Agilent Jet Stream (AJS) electrospray (ESI) source. The details of the lipidomic method are described elsewhere (10.3390/metabo12020124), briefly an Acquity BEH C18 column (1.7 μm, 2.1 mm × 150 mm; Waters, USA) was used for chromatographic separation. Lipids were separated by a gradient of mobile phases A: 10 mM ammonium formate and 0.1% formic acid in acetonitrile:water (60:40, v/v); mobile phase B was 10 mM ammonium formate and 0.1% formic acid in 2-propanol:acetonitrile (90:10, v/v) in ESI + mode, for ESI- mode formic acid and ammonium formate were replaced by acetic acid and ammonium acetate.

The details of metabolomic method are described in the supplement. Briefly, metabolites were separated on an Acquity HSS T3 column (1.7 μm, 2.1 mm × 100 mm; Waters, USA) using a gradient of mobile phases A: 5 mM ammonium formate and 0.1% formic acid in water:MeOH (95:5, v/v) and mobile phase B: 5 mM ammonium formate and 0.1% formic acid in MeOH in ESI + mode, for ESI- mode formic acid and ammonium formate were replaced by acetic acid and ammonium acetate. The mass spectrometer acquired data in Full MS and autoMS/MS modes in the *m/z* range of 50–1200.

#### Processing and statistical analysis of data generated by fingerprinting experiments

Regarding to the lipidomics data analysis, data were processed by the LipidMatch suite [26], which uses MZmine 2 for feature extraction and an R script for lipid identification. Lipids were identified based on fragmentation spectra and accurate mass in silico libraries, which are part of the LipidMatch suite. Fragmentation spectra of the significant compounds from metabolomics were also compared to those present in METLIN and LIPIDMAPS databases, and their identities were confirmed. In case of metabolomics data, the data were processed by Profinder software (Agilent Technologies, Santa Clara, CA, USA) and MZmine 2.

The data matrices obtained from lipidomics and metabolomics data processing were imported to SIMCA for multivariate analysis and Mass Profiler Professional (MPP, Agilent technologies for univariate analysis and metabolite profiling. Logarithmic transformation and pareto scaling were used to pre-process the data. Two-way ANOVA (corrected p-value cut-off: 0.05; p-value computation: Asymptotic; Multiple Testing Correction: Benjamini-Hochberg) analysis was applied to the data matrix to filter significant entities affected by diet group and sampling point factors.

### Sample size estimation

An a priori power analysis using GPOWER (Faul et al., 2007) with power set at 0.80 and significance level set at 0.05 was calculated retrospectively. The power analysis indicated that a total sample of 76 people would be needed to detect large effects (f = 0.40) for this study with 4 groups. A total sample of 180 people would be needed to detect medium effects (f = 0.25) Thus, the sample size was sufficient to reveal that a large effect could not be interpreted as non-significant.

## Results

### Parent study

Results for the primary outcome have been previously reported, showing that the VLCHF diet, either in isolation or in combination with HIIT, caused a significant reduction in body mass, visceral adipose tissue (VAT) mass and body composition variables. HIIT alone did not induce such effects on body composition, but improved CRF (Cipryan et al., 2021). We followed with a secondary analysis, which focused on cardiometabolic risk factors. The VLCHF diet was effective for favorable changes in the homeostasis model assessment of insulin resistance (HOMA-IR), Adiponectin/Leptin ratio and diastolic blood pressure. HIIT, or HIIT combined with the VLCHF diet, had no additional benefits for the analysed variables (Cipryan et al., 2022). Of note, total energy intake significantly decreased in the HIIT, VLCHF and VLCHF + HIIT groups. Carbohydrate intake significantly decreased and fat intake increased in the VLCHF and VLCHF + HIIT groups. Protein intake did not significantly change in any of the study groups (Cipryan et al., 2021).

### Body composition

VAT mass and body composition changes after 12 weeks significantly differed between study groups. VAT mass only decreased in the VLCHF and VLCHF + HIIT groups (p < 0.001, median [95% CI]: VLCHF: − 142.0 [− 187.0; − 109.5] g; VLCHF + HIIT: − 104.0 [− 135.0; − 71.0] g). Similarly, changes in body mass, total body fat, trunk fat mass, waist and hip circumferences were distinctly decreased in the VLCHF and VLCHF + HIIT groups, when compared to HIIT and Control groups. However, total lean mass significantly decreased in the VLCHF and VLCHF + HIIT groups (− 2.1 [− 3.0; − 1.6] kg and − 2.5 [− 3.6; − 1.8] kg, respectively), but not in HIIT and control groups, as presented in the parent study (Cipryan et al., 2021).

### Serum lipidomic analysis

#### Multivariate model analysis

**Fig. 1:**
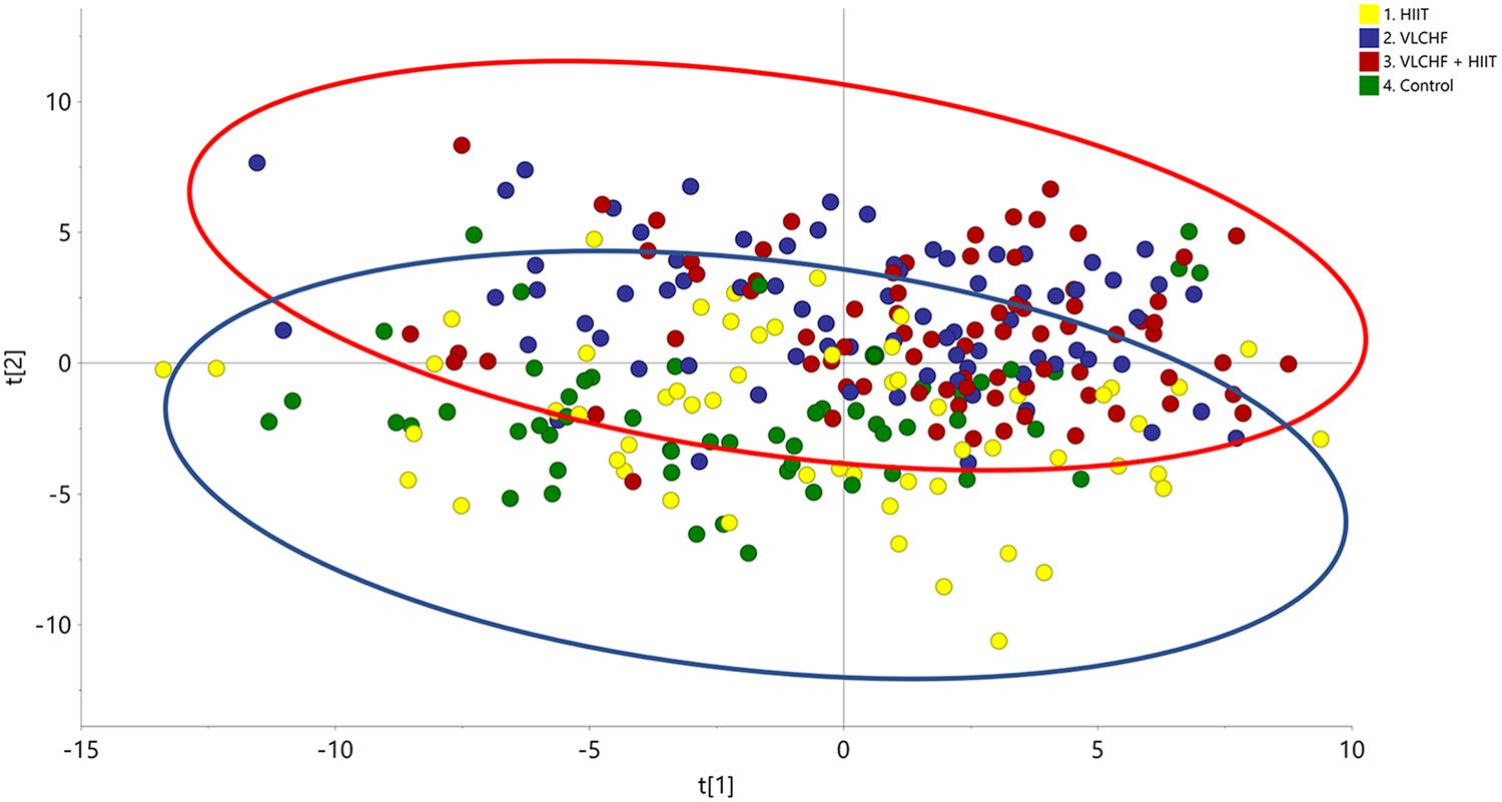
PCA plot of diet groups intervention including samples after 4, 8, and 12 weeks (T_1_, T_2,_ T_3_). Control, green label: control group; HIIT, yellow label: HIIT group; VLCHF, blue label: VLCHF diet group; VLCHF + HIIT, red label: VLCHF diet and HIIT group. Red ellipse: Samples grouped by VLCHF diet group; Blue ellipse: Samples grouped by non-VLCHF diet group

The pre-processing operations provided a data matrix based on 409 entities from the full data set. The data matrix was imported to SIMCA software for data processing including data log transformation and pareto scaling (van der Berg et al., 2006). A PCA model of the final data matrix was created by SIMCA software to describe the total variance of the full data set (Fig. 1). The calculated PCA model based on 267 samples described 84.9% of the variation in the data matrix (R2 X = 0.849) according to the cross-validation prediction of Q2 = 0.651. The PCA showed a global trend of the data variation describing a clear sample grouping affected by the VLCHF diet (VLCHF and VLCHF + HIIT groups; red circle, Fig. 1) and non-VLCHF diet (HIIT and Control groups; blue circle, Fig. 1). The results showed similar distributions of lipid compounds in both the VLCHF and VLCHF + HIIT groups and similar distributions of lipid compounds in both the HIIT only and the Control group.

#### Lipids identification and kinetic

**Fig. 2:**
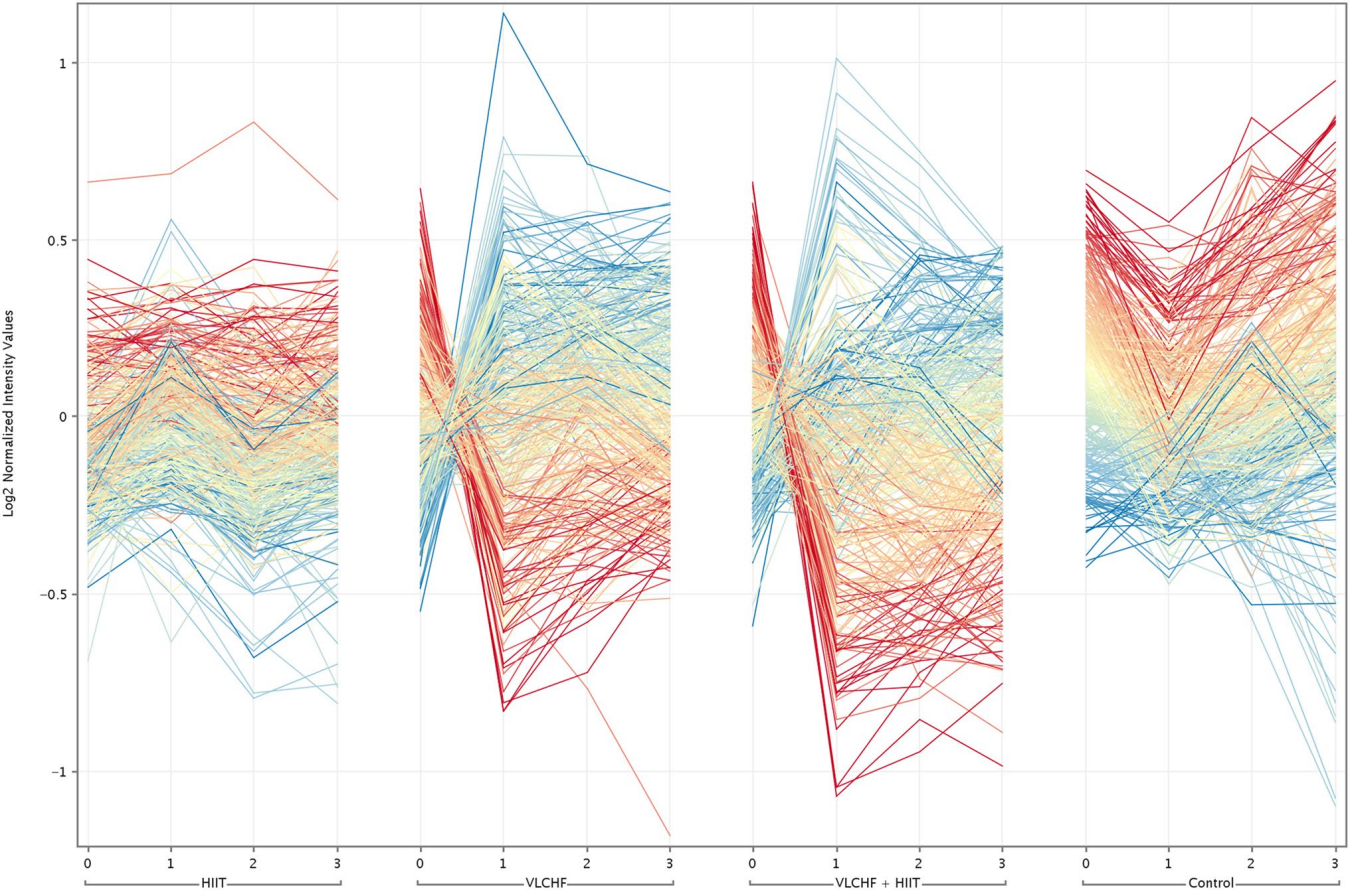
Profile plot of significantly changed lipids. Colored by changes respect to diet group at T_1_, T_2,_ T_3_. Red lines: Highly correlated with decreasing trend; Blue lines: Highly correlated with increasing trend; Yellow lines: low fold change according the trend

Two-way ANOVA (corrected p-value cut-off: 0.05) was applied to the data matrix in order to filter significant entities affected by diet groups and sampling point factors. The 146 entities were significant in both the VLCHF and VLCHF + HIIT groups (Fig. 2). The final list of entities was filtered using the correlation between the acquired values and parameter values (sampling point) in order to identify entities correlated with the increasing and decreasing trends along the study. This approach was carried out using Euclidean distance and Pearson correlation (correlation coefficient cut-off: 0.95).

The identified lipids were classified in 10 different families including plasmalogens (plasmanyl and plasmenyl), fatty acyl esters of hydroxy fatty acid (FAHFA), phosphatidylcholines (PC), phosphatidylethanolamines (PE), phosphatidylinositols (PI), Lysophsphatidylcholines (LPC), Lysophosphatidyletanolamines (LPE), dimethylphosphatidylethanolamines (DMPE), triglycerides (TG), fatty acids (FA), acetyl-L-carnitines (AcCar), cholesterol esters (CE) sphingomyelins (SM), ceramides (CER) and sulfated glycosphingolipids (sulfatide). The differences in trends of each lipid class could be clearly evaluated along the intervention groups as the members of the class almost uniformly followed the same pattern. Only in a minority of cases, different trends were observed for individual lipids compared to the other members of the class. One such example was Plasmenyl PS(P-20:1/20:4), which was downregulated in both dietary intervention groups but the rest of the lipid class family exhibited upregulation. The AcCars, plasmalogens, FAHFAs, SMs, CER, CE, FAs and sulfatide were mostly identified as increased markers in plasma after both the dietary and the training intervention. TGs and glycerophospholipids (PC, PE, PI and DMPEs) were mostly decreased lipids in serum after the VLCHF and VLCHF + HIIT interventions. In addition, AcCar, SMs, CER, CE, FAs and sulfatide were exclusively identified as families increased in the VLCHF and VLCHF + HIIT groups. PE, PI, LPE, LPC and DMPEs were exclusively identified as family decreased during the diet intervention.

Seventy-eight entities out of 146 were significantly unique for the VLCHF and VLCHF + HIIT groups (Fig. 2). No lipid compounds specifically influenced by exercise were identified (HIIT and Control groups).

Thirty-nine lipids increased in the VLCHF and VLCHF + HIIT groups **(**Table 1**)**. Plasmanyl-PC(O-16:0/20:5), Plasmanyl-PC(O-18:1/20:4) and SM(36:2) were the identified lipids with a highest increase in the VLCHF group after the 12 week intervention (T_3_). FAHFA(18:2/20:4), FAHFA(18:1/20:3) and SM(36:2) showed the highest variation after the first intervention month (T_1_) in the VLCHF group. Conversely, a total of 38 lipids with a decreasing trend were found in the VLCHF and VLCHF + HIIT groups and with stable trend in control and HIIT groups being principally triglycerides and glycerophospholipids (Table 2). FAHFA(14:0/22:3), PC(14:0/16:1) and PC(18:0/20:3) were the lipids with the greatest decrease identified in the VLCHF group after the 12 week intervention (T_3_). In addition to this, FAHFA(14:0/22:3) were the unique lipids that decreased during the entire 12 week study. PC(14:0/16:1), PC(14:0/18:2) and PC(18:0/20:3) showed the largest decrease after the first month of intervention (T_1_) in both VLCHF and VLCHT + HIIT groups.

**Table 1:** Serum lipids identified as significantly UP regulated biomarkers in the VLCHF and VLCHF + HIIT groups

ID	Compound	Family	Mass	Rt
1	AcCar(18:1)	Acetyl-L-carnitines*	426.3582	2.74
2	AcCar(18:0)	Acetyl-L-carnitines	428.3743	3.72
3	AcCar(16:0)	Acetyl-L-carnitines	400.3425	2.63
4	Plasmenyl-PE(P-16:0/20:4)	Plasmalogens	722.5112	9.44
5	Plasmenyl-PE(P-18:1/20:4)	Plasmalogens	748.5268	9.48
6	Plasmenyl-PE(P-18:0/20:4)	Plasmalogens	750.5397	9.28
7	Plasmanyl-PE(O-16:0/22:5)	Plasmalogens	750.5407	9.67
8	Plasmenyl-PE(P-18:0/20:4)	Plasmalogens	750.5423	10.35
9	Plasmanyl-PE(O-18:0/20:4)	Plasmalogens	752.5572	10.54
10	Plasmenyl-PE(P-18:0/20:3)	Plasmalogens	752.5577	10.91
11	Plasmenyl-PE(P-18:0/22:6)	Plasmalogens	774.5422	10.02
12	Plasmenyl-PE(P-18:1/22:5)	Plasmalogens	774.5429	9.43
13	Plasmenyl-PE(P-18:0/22:5)	Plasmalogens	776.5592	10.31
14	Plasmanyl-PE(O-18:0/22:5)	Plasmalogens	778.5755	10.5
15	Plasmenyl-PE(P-20:0/20:4)	Plasmalogens	778.5747	11.16
16	Plasmenyl-PE(P-20:0/22:6)	Plasmalogens	802.5748	10.91
17	Plasmanyl-PC(O-16:0/18:2)	Plasmalogens	744.589	9.21
18	Plasmanyl-PC(O-16:0/20:5)	Plasmalogens	766.5751	8.91
19	Plasmanyl-PC(O-16:0/20:4)	Plasmalogens	768.5895	9.05
20	Plasmenyl-PC(P-16:0/22:6)	Plasmalogens	790.573	8.65
21	Plasmenyl-PC(P-18:1/20:4)	Plasmalogens	792.5887	8.93
22	Plasmanyl-PC(O-18:1/20:4)	Plasmalogens	794.6042	9.64
23	Plasmanyl-PC(O-20:1/18:3)	Plasmalogens	796.6189	9.57
24	Plasmanyl-PC(O-18:0/20:4)	Plasmalogens	796.6202	9.77
25	Plasmanyl-PC(O-18:1/22:6)	Plasmalogens	818.6037	8.81
26	Plasmanyl-PC(O-20:1/20:4)	Plasmalogens	822.6356	9.74
27	FAHFA(18:2/20:4)	Fatty acyl esters of hydroxy fatty acid	581.4536	3.94
28	FAHFA(18:1/20:3)	Fatty acyl esters of hydroxy fatty acid	585.4851	5.06
29	FAHFA(18:1/22:3)	Fatty acyl esters of hydroxy fatty acid	613.5216	5.06
30	SM(d17:1/16:0)	Sphingomyelin*	689.5589	8.15
31	SM(d18:0/16:0)	Sphingomyelin	705.5893	8.87
32	SM(d17:1/18:0)	Sphingomyelin	717.5896	8.98
33	SM(36:2)	Sphingomyelin	787.5947	8.88
34	SM(d18:1/18:0)	Sphingomyelin	731.6059	9.38
35	SM(36:0)	Sphingomyelin	791.6243	10.10
36	HexCer-NS(d-18:1/23:0)	Ceramides*	796.6645	12.14
37	CE(20:4)	Cholesterol ester*	695.5738	13.52
38	FA(18:1)	Fatty acids*	281.2488	5.06
39	Sulfatide(39:5)	Sulfatide	840.5304	8.54

*Family exclusively identified in the group of lipids that increase

**Table 2:** Serum lipids identified as significantly DOWN regulated biomarkers in the VLCHF and VLCHF + HIIT groups

ID	Compound Name	Family	Mass	Rt
1	Plasmenyl-PS(P-20:1/20:4)	Plasmalogens	820.546	9.19
2	FAHFA(14:0/22:3)	Fatty acyl esters of hydroxy fatty acid	559.4694	8.49
3	TG(12:0/16:0/18:1)	Triglycerides	794.7225	13.27
4	TG(12:0/18:2/18:2)	Triglycerides	816.7069	12.49
5	TG(14:1/16:0/18:3)	Triglycerides	816.707	12.60
6	TG(16:1/16:1/16:1)	Triglycerides	823.6784	12.87
7	TG(14:0/16:1/18:1)	Triglycerides	820.7392	13.27
8	TG(14:0/16:0/18:1)	Triglycerides	822.7542	13.65
9	TG(14:0/18:2/18:3)	Triglycerides	842.7226	12.62
10	TG(14:0/18:2/18:2)	Triglycerides	849.6939	13.09
11	TG(16:0/16:0/18:4)	Triglycerides	849.694	12.91
12	TG(16:0/16:1/18:3)	Triglycerides	844.7387	12.92
13	TG(14:0/16:0/20:4)	Triglycerides	844.7389	13.13
14	TG(14:0/18:1/18:2)	Triglycerides	846.7545	13.28
15	TG(16:0/16:0/18:3)	Triglycerides	851.7099	13.26
16	TG(16:1/18:2/18:3)	Triglycerides	868.7381	12.74
17	TG(16:0/18:3/18:3)	Triglycerides	873.6943	12.73
18	TG(16:0/18:2/18:3)	Triglycerides	870.7541	12.97
19	PC(28:0)	Glycerophospholipids	700.4884	7.73
20	PC(14:0/16:1)	Glycerophospholipids	762.5306	7.91
21	PC(14:0/16:0)	Glycerophospholipids	706.5383	8.53
22	PC(14:0/18:2)	Glycerophospholipids	730.538	7.95
23	PC(16:0/16:1)	Glycerophospholipids	732.553	8.61
24	PC(14:0/20:4)	Glycerophospholipids	754.5367	7.79
25	PC(34:3)	Glycerophospholipids	778.536	8.31
26	PC(17:1/18:2)	Glycerophospholipids	770.5686	8.59
27	PC(14:0/22:6)	Glycerophospholipids	836.5411	7.62
28	PC(18:2/18:3)	Glycerophospholipids	780.5529	7.58
29	PC(16:0/22:4)	Glycerophospholipids	810.5981	8.98
30	PC(18:0/20:3)	Glycerophospholipids	812.6163	9.64
31	PC(18:0/22:5)	Glycerophospholipids	836.6115	9.61
32	PE(18:0/20:3)	Glycerophospholipids	768.5526	10.31
33	LPC(14:0)*	Glycerophospholipids	468.3088	2.06
34	LPC(16:1)	Glycerophospholipids	494.3241	2.16
35	LPC(20:3)	Glycerophospholipids	546.3549	2.50
36	DMPE(16:0/16:1)	Glycerophospholipids	716.5225	8.83
37	DMPE(16:0/20:3)	Glycerophospholipids	768.5523	9.20
38	DMPE(18:0/20:3)	Glycerophospholipids	796.5873	10.13

*PE, LPC and DMPE were exclusively identified in the group of lipids that decrease

### Serum metabolomic analysis

#### Multivariate model analysis

The multivariate model analysis procedures were performed under the same criteria as lipidomics. The pre-processing operations gave in case of metabolomics analysis a data matrix based on 5618 entities from full data set.

These results showed similar intervention group distributions as lipidomics. The metabolites in plasma samples showed a clear sample grouping, i.e. (VLCHF and VLCHF + HIIT groups) vs. (HIIT and Control groups).

#### Metabolites identification and kinetic

Two-way ANOVA (corrected p-value cut-off: 0.05; p-value computation: Asymptotic; Multiple Testing Correction: Benjamini-Hochberg) statistics analysis was applied to the data matrix in a similar manner as in lipidomics. A total of 164 of these metabolites satisfying the filtration criteria for up and down regulation were phospholipids already identified and described in the previous lipidomics analysis. A total of 8 entities were exclusively identified in the metabolomics analysis and these entities significantly increased in the VLCHF and VLCHF + HIIT groups after 12 weeks (T_3_).

The metabolites identified tentatively were classified in 4 families including glycerophospholipids, xanthine, acyl-carnitine and alpha hydroxy acids. The glycerophospholipids and xanthine were identified as metabolites that decrease in the VLCHF and VLCHF + HIIT groups. The acyl-carnitines and alpha hydroxyl acids were identified as metabolites that increased in the VLCHF group during the 12 week study (T_3_).

Regarding to the individual kinetics of the metabolites identified, a total of 4 metabolites were found with an increasing trend in the VLCHF and VLCHF + HIIT groups along the 12 week study and without a defined trend in the HIIT and the Control group (Table 3). Decanoylcarnitine, 2-Decenoylcarnitine, Octanoylcarnitine and 4-Hydroxybutyric acid were found according with an increased trend in both VLCHF and VLCHF + HIIT groups. In the opposite, a total of 4 metabolites including three isomers of PE-NMe (18:1/18:3) and theobromine were found according with a decreased trend in the VLCHF and VLCHF + HIIT groups. Acyl-carnitines, hydroxybutiric acid and xanthine, tentatively identified by untargeted metabolomics, were confirmed by MS/MS spectra fragmentation identification therefore increase their identification accuracy to level 2 (Table 3) (Sumner et al., 2007).

**Table 3:** Significantly changed plasma metabolites in the VLCHF and VLCHF + HIIT groups identified by untargeted metabolomics

ID	Compound name	Regulation	MS/MS fragment	Mass	Rt
1	Decanoylcarnitine^1^	UP	316.2465,257.1732,155.1413,144.1031	315.2405	13.50
2	2-Decenoylcarnitine^1^	UP	314.231,85.0279,255.1586,297.1568^*^	313.2249	12.66
3	Octanoylcarnitine^1^	UP	288.2163,229.1436,85.0286,127.1131^*^	287.209	11.23
4	4-Hydroxybutyric^1^	UP	103.0398, 57.0332	104.0468	1.12
5	PE-NMe(18:1/18:3)^2^	DOWN	–	753.5298	20.01
6	PE-NMe(18:1/18:3)^2^	DOWN	–	753.5309	20.01
7	PE-NMe(18:1/18:3)^2^	DOWN	–	753.5299	20.01
8	Theobromine^1^	DOWN	181.0714,138.0656,110.0712,163.0630	180.0636	4.42

^*^Collision energy of 10 eV

^1^Identification level 2 by MS/MS according with Sumner et al. (Sumner et al., 2007)

^2^Identification level 3 by exact mass according with Summer et al. (Sumner et al., 2007)

## Discussion

This is the first randomized control trial which investigated the effects of a 12 week VLCHF diet and HIIT interventions, as well as their combination, on changes of plasma lipidomic and metabolomic profiles in individuals with overweight and obesity. We revealed that the VLCHF diet, alone or combined with HIIT, caused a substantial increase in serum plasmalogens, FAHFAs, FAs, AcCar, CE, SMs, CER, and sulfatides. Glycerophospholipids and TGs substantially decreased in both the VLCHF and VLCHF + HIIT groups. The highest lipid changes occurred already after 4 weeks and then their plasma levels remained stable or showed a countermovement trend until the end of the intervention after 12 weeks (Fig. 2). Similar to lipidomic, the metabolomic analysis revealed differences between the groups including the diet (i.e. VLCHF and VLCHF + HIIT) versus the HIIT and Control group. Four metabolites were significantly upregulated and four were downregulated in the VLCHF and VLCHF + HIIT groups. HIIT alone had no substantial changing effect on lipidomic and metabolomic markers investigated although data were more stable than compared to the control group which showed contrary trends to both diet groups. This may indicate a stabilizing effect of exercise on lipid and metabolic markers.

### Plasmalogens

We revealed a significant increase in plasmalogens (Table 1). Plasmalogens are a class of glycerophospholipids, reflect the functional activity of peroxisomes and have been reduced (or deficient) in chronic diseases related to oxidative stress, chronic inflammation and aging, such as Parkinson disease, Alzheimer disease, metabolic syndrome and type 2 diabetes mellitus (Bozelli et al., 2021; Braverman & Moser, 2012; Kytikova et al., 2020) and their levels are altered also in various types of cancers (Messias et al., 2018). Despite the biological mechanism of action for plasmalogens remains unclear, it seems that plasmalogens play a crucial role as endogenous antioxidants, protecting other phospholipids, lipids and lipoprotein particles from oxidative stress, as well as participate in PUFA cell metabolism and play an important role as neuroprotectors and modulators of the signalling mechanisms realized in cell membrane (Hossain et al., 2016; Kytikova et al., 2020).

It has been shown that a plasmalogen replacement therapy can be a successful anti-inflammatory strategy, which ameliorates several pathological hallmarks of neurodegenerative diseases, atherosclerosis, insulin resistance and hepatic steatosis (Bozelli et al., 2021; Paul et al., 2019), alleviates negative mood states and sleep problems and enhance mental concentration (Fujino et al., 2022). Some plasmalogens subtypes levels (ethanolamine) are decreased in the Alzheimer´s disease patients and this lower level correlates with cognition deficit and severity of the disease (Su et al., 2019). Therefore, a therapeutic modulation of plasmalogens can be a promising tool in prevention and treatment of several chronic diseases. For example, supplementation with alkylglycerol, e.g. abundant in shark liver oil, bypass the peroxisome because alkylglycerol can be incorporated directly into the phospholipid pathway, which increases plasmalogen biosynthesis, similarly as a consumption other marine foodstuff (particularly blue mussel and ascidian). This plasmalogen enrichment may provide protection against obesity-related dyslipidaemia and inflammation (Paul et al., 2019, 2021). We showed for the first time that plasmalogens can be increased by a VLCHF diet, which can serve as a potential mechanistic co-explanation of some beneficial effects of such a VLCHF diet on chronic diseases (Silverii et al., 2022).

### FAHFAS

Fatty acid esters of hydroxyl-fatty acids (FAHFAs) are another extensive group of lipids, which increased after the 12 week VLCHF diet. Since Yore et al. (Yore et al., 2014) reported in 2014 the discovery of the FAHFAs, there is still very few human data available and their biological effects are not well understood. FAHFAs have been found in different foods. Animal products contain more FAHFAs compared to plant products (Yore et al., 2014) and it seems that nutrition has a predominant impact on FAHFA level in serum (Kellerer et al., 2021). As the VLCHF diet is typically abundant in animal food sources, it is not surprising that we have found a FAHFA serum increase.

FAHFAs can also be incorporated in triacylglycerols (TG) as one of the three fatty acids. FAHFA-TGs are major reservoir of FAHFAs in cells and tissues (Tan et al., 2019) and, therefore, the nutrition impact on the FAHFA level in serum may not be exclusive. Also, the FAHFA levels are increased when lipolysis is induced (Tan et al., 2019) which is the elementary underpinning of the VLCHF diet (i.e. shifting from carbohydrate to predominant lipid metabolism).

There is mounting evidence that type 2 diabetes can be reversed through carbohydrate restricted diets (Goldenberg et al., 2021; Gram‐Kampmann et al., 2022; Joseph et al., 2022; Skow & Jha, 2022; Volek et al., 2021), however, the exact mechanism on a cellular level is not clear. Since FAHFAs show anti-inflammatory and anti-diabetic effects (Kellerer et al., 2021; Riecan et al., 2022; Tan et al., 2019; Yore et al., 2014), our finding of increased FAHFA levels after a 12 week VLCHF diet may contribute to this explanation.

### Acetyl-L-Carnitines

Another upregulated lipid group after the 12 week VLCHF diet was Acetyl-L-Carnitines (ALC). ALC is a natural derivate of carnitine and can be found in various mammalian tissues, particularly in liver and skeletal muscles. Carnitine and its derivates play an essential role in the intermediary metabolism of glucose and β-oxidation of long-chain fatty acids. As a widely diffused dietary supplement, ALC may have a potential for the prevention of the oxidative stress (Lopez-Maldonado et al., 2021).

It has been shown in animal models that ALC inhibits the development of atherosclerosis by regulating blood lipids and inhibiting the gene expression of inflammatory factors and oxidative stress (Wang et al., 2020). This ALC anti-inflammatory and anti-angiogenic properties probably cause also the capability of ALC to down-modulate growth, adhesion, migration and invasion of prostate cancer cells (Baci et al., 2019). Bene et al. reviewed that ALC improves neurophysiological parameters, reduces pain and vascular-related symptoms in diabetic patients (Bene et al., 2018). ALC crosses the blood–brain barrier and improves neuronal energetic and repair mechanisms. It is therefore of great interest for its wide clinical application in various neurological disorders (e.g. Alzheimer’s dementia or depression) as well as HIV infection, chronic fatigue syndrome, peripheral neuropathies, ischemia and reperfusion of brain (Malaguarnera, 2012). Animal models showed that ALC supplementation ameliorates cognitive disabilities and combats oxidative stress-induced neuroinflammation (Verma et al., 2021). Beside such promising effects, the potential use of ALC supplementation in the pharmacotherapy of Alzheimer disease is still under debate (Pennisi et al., 2020). In contrast to these possible benefits, there are also some indices that L-carnitine-related metabolites can increase the risk for cardio-metabolic disease (Bene et al., 2018).

### Oleic acid

We showed substantial increase of serum oleic acid (OA) (18:1)) which is a monounsaturated fatty acid and major fatty acid in dietary fat and in plasma triglycerides. OA-rich diet is associated with improved body mass and body composition. Particularly, abdominal fat and central obesity can be reduced following consumption of high-OA-containing meals (Tutunchi et al., 2020). OA is the most present fatty acid in olive oil, where it accounts for 58.5–83.2% of all methyl esters (Boskou et al., 2006). Olive oil is an important part of the Mediterranean diet, which is considered a healthy dietary pattern (US Department of Agriculture, 2020). Since we recommended to increase the fat intake including olive oil within the VLCHF diet in this study, it might be an explanation of the increased OA concentration found in serum of the diet group subjects. However, an alternative explanation can be based just on an increased lipolysis in adipose tissue and releasing OA into the blood, since the OA is the most abundant fatty acid in adipose tissue (Berry et al., 1986; Hellmuth et al., 2013).

The consumption of olive oil has been associated with several health benefits. However, it is unclear whether these beneficial effects are related to its polyphenol contents, OA or to the combination of them. It seems that consumption of olive oil or OA is as good as other strategies for Metabolic Syndrome management (Pastor et al., 2021). OA has also a potential role in controlling insulin resistance and type 2 diabetes mellitus, improving mitochondrial function, endothelial function, β-cell function, glucolipotoxicity, inflammation, oxidative stress, apoptosis, etc. (Rehman et al., 2020). However, studies of OA have reported contradictory results. For example, it has been reviewed that OA supplementation causes a significant reduction in blood concentrations of CRP in individuals with higher baseline level but has no significant effect on other inflammatory markers (Wang et al., 2022).

### Sphingomyelin and ceramide

Sphingomyelin and ceramide were other upregulated lipids in the VLCHF and VLCHF + HIIT groups. Sphingolipids are a large class of lipids with diverse functions in many (patho-) physiological processes. Sphingomyelin (SM) is the most abundant sphingolipid in the cell. It is one of the major components of the plasma membrane. The hydrolysis of SM increases the concentration of ceramide (Cer), a bioactive molecule, which is involved in cellular proliferation, growth and apoptosis. SM is essential for brain development and cognitive abilities and it is altered in neurodegeneration. Disturbances of enzymes involved in the SM cycle (i.e. ceramide/SM balance system) were observed in many CNS pathologies, including ischemia, Alzheimer disease, Parkinson disease, depression or schizophrenia (Bienias et al., 2016; Signorelli et al., 2021). It appears that increasing ceramide/SM ratio is important in the suppression of tumour growth (Taniguchi & Okazaki, 2021). Nevertheless, the specific role of the diverse SM species in plasma membrane are not well understood. It has been shown that an increased serum SM is associated with renal and coronary heart disease in type 1 diabetes (Pongrac Barlovic et al., 2020) and palmitoyl SM is significantly associated with cardiovascular risk in a Chinese population with type 2 diabetes (Chen et al., 2022). Conversely, an inverse association between SM (32:1) and incident ischemic stroke independent of established cardiovascular risk factors was identified in Swedish cohorts (Lind et al., 2020). SM from food, particularly milk and dairy products, are increasingly recognized as bioactive lipids but there is a lack of clinical trials data about role of SM intake (Jiang et al., 2022).

Cer is a central molecule among the various sphingolipids because it is involved in synthesizing complex structural sphingolipids (sphingomyelin and ganglioside) and as a precursor of sphingosine-1-phosphate (S1P). Cer has been recognized a cause of selective insulin resistance and dyslipidaemia. Increased serum Cer levels are strongly associated with adverse CV risks and events. Therefore, decreasing ceramide levels through pharmacology and gene modification of the enzymes involved in the synthesis and degradation of ceramide can be a potential strategy to combat CVDs (Shu et al., 2022; Tippetts et al., 2021). Plasma Cers are elevated in humans with early coronary atherosclerosis and coronary endothelial dysfunction (Akhiyat et al., 2022). De novo synthesis pathway of Cers in hepatocytes has been shown to exacerbate non-alcoholic fatty liver disease (Yu & Wang, 2022).

The increased serum Cer level in our study might be related to the marked increase in fat intake in the VLCHF diet groups. When FA availability exceeds the cell’s storage and energetic capacities, Cers and other intermediates in these biosynthetic pathways start to accrue. Further, Cers initiate actions to enable storage or utilization of FAs to help the cell adapt to the surplus of FAs and protect membrane bilayers (Tippetts et al., 2021). Recent evidence has shown that lipids, particularly saturated FA (SFA), can interfere with de novo Cer synthesis and hypothalamic regulatory disturbance. However, this usually happens in response to excessive consumption of SFA associated with dietary simple sugars. While Cers are associated with hypothalamic dysfunction in energy balance and may play a role in obesity development, S1P seems to be a central satiety factor in the hypothalamus (Reginato et al., 2021), which can contribute to an explanation of a typical higher satiety in the VLCHF diet.

We showed also a significant increase of sulphated glycosphingolipids (GSLs). However, there is still limited knowledge about their biological function. The sulphated GSLs play an important role in cell–cell communication (Honke et al., 2004) and it seems that they enhance apoptotic cell clearance and modulate macrophage activity within tumours (Popovic et al., 2007).

### Metabolomic outcomes

Decanoylcarnitine and octanoylcarnitine are members of the class of acylcarnitines with a general role to transport acyl-groups from the cytoplasm into the mitochondria for β-oxidation. Increased or decreased levels of these medium-chain fatty acids are associated with many diseases but without clear mechanistic explanations (Dambrova et al., 2022). However, we attribute the presented decanoylcarnitine and octanoylcarnitine, as well as short-chain fatty acid 4-hydroxybutyric, upregulations to increased fatty acids metabolism in the VLCHF and VLCHF + HIIT groups.

The downregulation of glycerophospholipid PE-NMe (18:1/18:3) (Table 3) and other triglycerides and glycerophospholipids within the lipidomic analysis (Table 2) is an expected response which may occur in carbohydrate intake restriction (Gjuladin-Hellon et al., 2019). This effect is explained by increased dependence on fat as a metabolic substrate which causes its increased peripheral uptake (Norwitz et al., 2022).

We showed a decrease of theobromine, a methylxanthine alkaloid derivate which is highly presented in cocoa in the VLCHF and VLCHF + HIIT groups. An animal study shows a theobromine beneficial role in body mass gain and effects on lipid and glucose metabolism (Camps-Bossacoma et al., 2019). There is an isolated evidence that theobromine may improve non-alcoholic fatty liver disease by inhibiting lipogenesis and fatty acid uptake and promoting fatty acid oxidation in the liver and hepatocytes (Wei et al., 2021). However, the theobromine role in human metabolism remains vague and do not allow to make a strong evidence-based conclusion about the presented outcomes.

### Strength and limitations

We consider the main strength of this study showing another research perspective for the VLCHF and HIIT issue which can contribute to a better understanding the effects of such a diet with and without exercise. The lipidomic and metabolomic results complement the performance, body composition and biochemical data which have been already presented elsewhere (Cipryan et al., 2021, 2022). Plasma lipidomic profiles may contribute to a better prediction of cardiovascular (CV) risk and events. Sphingolipids, phospholipids (including lyso- and ether- species), cholesteryl esters and glycerolipids were associated with future CV events and CV deaths in elderly with type 2 diabetes mellitus and more than one additional CV risk factors (Alshehry et al., 2016).

The limitations of the study design have been discussed previously (Cipryan et al., 2021, 2022). Specifically to these results, plasmalogen levels change as a function of human age (Bozelli et al., 2021). We have to consider this fact since the participant’s age was substantially heterogenous in all four study groups. Additionally, the long-term dietary intervention studies are likely to cause modulation of the gut microbiota. The modulations of the microbiota would modify the final pool of secondary bile acids by altering for example the bacterial Bile salt Hydrolase (BSH) enzyme activity and producing an alteration of the presence of total free bile acids. This modification might affect the interaction with the specific intestinal receptors as Farnesoid X receptor (FXR) and affect to the regulation of the lipids metabolism (Garcia et al., 2022; Lei et al., 2022; Porez et al., 2012). Also, we need to consider a possible interaction of the presented lipidomic and metabolomic outcomes with body composition changes after 12 weeks.

We have demonstrated the metabolomic and lipidomic differences between the VLCHF and VLCHF + HIIT groups versus HIIT and control groups along with a significant total energy decrease in all intervention groups (except the control group) (Cipryan et al., 2021). Therefore, we presume that the presented changes are not the total energy decrease related.

## Conclusions

In addition to a significant loss of body fat and body mass, the VLCHF diet affected plasma lipids considerably while the effect of HIIT was insignificant, although HIIT alone obviously stabilized the profiles compared to all other groups including controls. We showed substantial changes of plasma lipids already after 4 weeks of intervention in the participants in the VLCHF and VLCHF-HIIT groups which might correspond to the enhancement of metabolic flexibility to use lipid compounds as a substrate for energy metabolism. These changes persisted throughout the entire 12 week of both VLCHF diet interventions. We did not observe any synergistic effect of the VLCHF diet and HIIT on lipidomic and metabolomic profiles. Specifically, acyl carnitines, plasmalogens, fatty acyl esters of hydroxy fatty acid, sphingomyelin, ceramides, cholesterol esters, fatty acids, and 4-hydroxybutyric were identified as lipid families that increased in the VLCHF diet groups whereas lipid families of triglycerides and glycerophospholipids decreased. Additionally, metabolomic analysis showed a decrease of theobromine.

### Supplementary Information

Below is the link to the electronic supplementary material.

Supplementary file1 (DOCX 368 kb)

## Data Availability

Data described in the manuscript, code book, and analytic code will be made available upon request pending approval by authors.
