# Supplementary material for: A lipidomic and metabolomic signature of a very low-carbohydrate high-fat diet and high-intensity interval training: an additional analysis of a randomized controlled clinical trial
Source: Metabolomics. 2023 Dec 23;20(1):10. doi: 10.1007/s11306-023-02071-1 (PMC10748768; doi:10.1007/s11306-023-02071-1)
Supplement: Supplementary file 1 — Supplementary file1 (DOCX 368 kb) [file 11306_2023_2071_MOESM1_ESM.docx]

**Supplementary Material**

**Table S1. Chromatographic gradient for metabolomic method for both ionization polarities**

| time [min] | Flow [µL/min] | A [%]* | B [%]** |
| --- | --- | --- | --- |
| 0 | 0,4 | 99 | 1 |
| 2 | 0,4 | 99 | 1 |
| 18 | 0,4 | 1 | 99 |
| 23,5 | 0.5 | 1 | 99 |
| 23,6 | 0.4 | 99 | 1 |
| 25 | 0.4 | 99 | 1 |

* 5 mM ammonium formate and 0.1% formic acid in water:MeOH (95:5, v/v) in ESI+ and * 5 mM ammonium acetate and 0.1% acetic acid in water:MeOH (95:5, v/v) in ESI-
** 5 mM ammonium formate and 0.1% formic acid in MeOH in ESI+ 5 mM ammonium acetate and 0.1% acetic acid in MeOH in ESI-

**Table S2. Chromatographic gradient for lipidomic method in ESI+.**

| time [min] | Flow [µL/min] | A [%]* | B [%]** |
| --- | --- | --- | --- |
| 0 | 0.3 | 60 | 40 |
| 2 | 0.3 | 60 | 40 |
| 4 | 0.3 | 50 | 50 |
| 5 | 0.3 | 40 | 60 |
| 15 | 0.3 | 0 | 100 |
| 15.1 | 0.3 | 60 | 40 |
| 17 | 0.3 | 60 | 40 |

* 10 mM ammonium formate and 0.1% formic acid in acetonitrile:water (60:40, v/v)
** 10 mM ammonium formate and 0.1% formic acid in 2-propanol:acetonitrile (90:10, v/v)

**Table S3. Chromatographic gradient for lipidomic method in ESI-.**

| time [min] | Flow [µL/min] | A [%]* | B [%]** |
| --- | --- | --- | --- |
| 0 | 0.3 | 60 | 40 |
| 2 | 0.3 | 60 | 40 |
| 4 | 0.3 | 50 | 50 |
| 5 | 0.3 | 40 | 60 |
| 12 | 0.3 | 20 | 80 |
| 12.1 | 0.3 | 0 | 100 |
| 15 | 0.3 | 0 | 100 |
| 15.1 | 0.3 | 60 | 40 |
| 18 | 0.3 | 60 | 40 |

* 10 mM ammonium acetate and 0.1% acetic acid in acetonitrile:water (60:40, v/v)
** 10 mM ammonium acetate and 0.1% acetic acid in 2-propanol:acetonitrile (90:10, v/v)

**Random Assignment**

Participants who successfully met inclusion criteria become acquainted with all study requirements. Those who agreed to follow were eligible for randomization. The randomization was stratified by age (20-29, 30-39, 40-49, 50-59 years) and sex (male, female). A set of blinded envelopes with study group number (1-4) were prepared by a principal researcher. To ensure close balance among four study groups at any point in the study. The study group assignments were randomly permuted within blocks of 8 participants (2 participants randomly allocated to each study arm). A specific ID were created for each participant and the key was kept accessible only to staff responsible for data collection.

**Table S4. Baseline characteristics of study participants.**

|  | **HIIT**  (N=22) | **VLCHF**  (N=25) | **VLCHF+HIIT**  (N=25) | **Control**  (N=19) |
| --- | --- | --- | --- | --- |
| Male:Female | 6:16 | 8:17 | 7:18 | 6:13 |
| Age (year) | 46 (38.8; 53.3) | 43 (35.0; 51.5) | 43 (32; 51) | 40 (31; 53) |
| Height (cm) | 167.7 (160.6; 175.0) | 170.1 (164.7; 177.9) | 169.8 (160.9; 179.7) | 171.9 (162.8; 177.6) |
| BMI (kg.m^-2^) | 28.7 (26.9; 30.9) | 31.3 (27.7; 33.0) | 31.0 (27.2; 35.1) | 28.7 (26.7; 32.9) |
| WHtR (-) | 0.60 (0.54; 0.62) | 0.62 (0.56; 0.65) | 0.62 (0.54; 0.68) | 0.60 (0.54; 0.63) |

Legend: BMI – body mass index, WHtR – waist-to-height ratio.

Values are shown as median (lower and upper quartile).


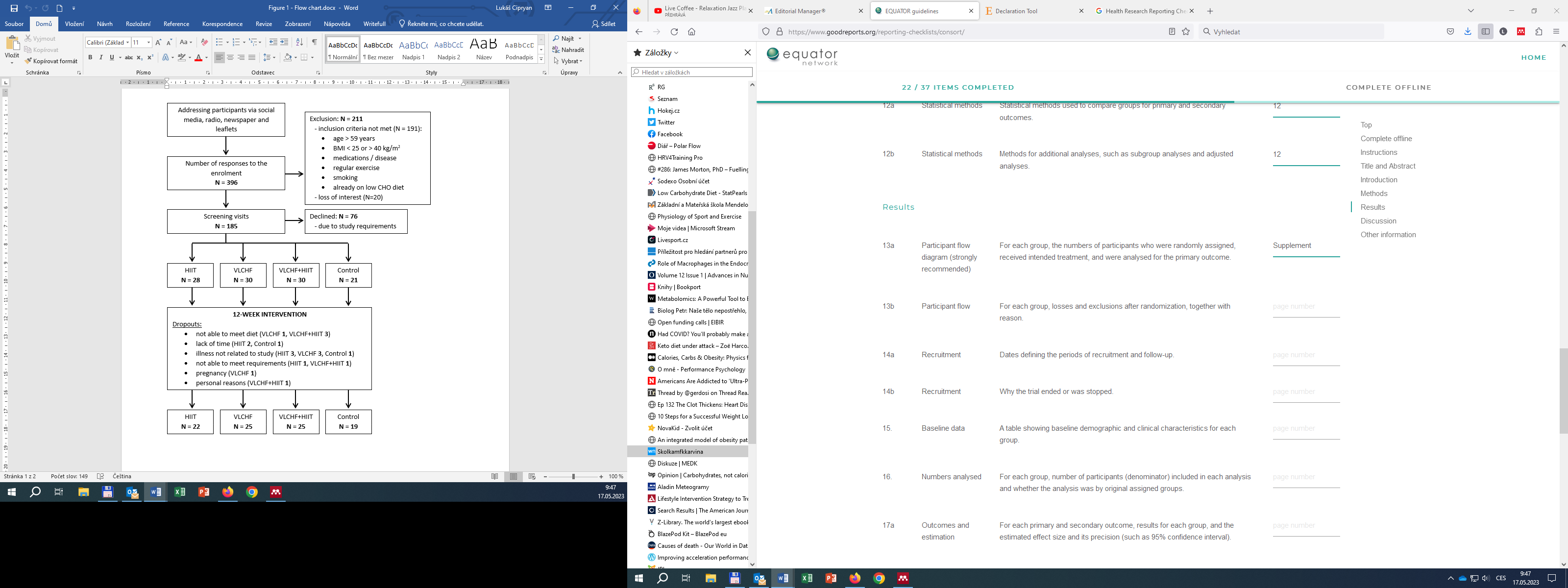


**Figure S1. Flow chart.**
